# Ningmitai capsules have anti-inflammatory and pain-relieving effects in the chronic prostatitis/chronic pelvic pain syndrome mouse model through systemic immunity

**DOI:** 10.3389/fphar.2022.949316

**Published:** 2022-10-03

**Authors:** Hanchao Liu, Zhenqing Wang, Qigen Xie, Ani Chi, Yanqing Li, Jian Dai, Min Zhang, Chunhua Deng, Guihua Liu

**Affiliations:** ^1^ Department of Andrology, The First Affiliated Hospital of Sun Yat-sen University, Guangzhou, China; ^2^ Department of Pediatric Surgery, The First Affiliated Hospital of Sun Yat-sen University, Guangzhou, China; ^3^ Reproductive Medicine Research Center, The Sixth Affiliated Hospital of Sun Yat-sen University, Guangzhou, China

**Keywords:** Ningmitai capsules, chronic prostatitis/chronic pelvic pain syndrome, anti-inflammation, pain-relieving, systemic immunity

## Abstract

Chronic prostatitis/chronic pelvic pain syndrome (CP/CPPS) seriously affects the physical and mental health of approximately 90% of males. Due to its complex and unclear etiology, the treatment methods that are currently available for chronic prostatitis/chronic pelvic pain syndrome are controversial, and their efficacy is unsatisfactory. At present, most researchers believe that this kind of prostatitis is caused by autoimmune inflammation. Chinese herbs, which are the essence of traditional Chinese medicine (TCM), are emerging treatment options for inflammation and immune diseases. In this experiment, we investigated the effect of Ningmitai capsules (a kind of traditional Chinese medicine widely used to treat lower urinary tract inflammation and pain in males) on chronic prostatitis/chronic pelvic pain syndrome in a non-obese diabetes-experimental autoimmune prostatitis (NOD-EAP) mouse model. First, by using bioinformatics analysis of data from the Encyclopedia of Traditional Chinese Medicine (ETCM) database, we found that quercetin, which is one of the main components of Ningmitai capsules, could reduce the secretion of CCL2 by inhibiting the MAPK pathway. In animal experiments, it was found that after Ningmitai treatment, the inflammation in mouse prostates was alleviated, the expression of CCL2, which is related to pain, and MAPK pathway components were downregulated, and the activation of the inflammatory NF–κB and STAT3 pathways was reduced. Pelvic pain and inflammation were relieved in mice with EAP. Due to the presence of the blood–prostate barrier, the drug may not completely reach the prostate directly and take effect locally. However, we found that after Ningmitai treatment, the proportions of proinflammatory CD11b^+^Ly6C^high^ immune cells in the spleen, bloodstream (systemic immunity), and prostate (local immunity) were reduced. The infiltration of CD11b^+^ immune cells into the spleen and prostate was decreased. These findings suggested that Ningmitai can treat chronic prostatitis/chronic pelvic pain syndrome by affecting systemic and local immunities through the CCL2–MAPK pathway.

## Introduction

Chronic prostatitis is the most common chronic inflammatory disease in men, and it seriously affects the physical and mental health of patients (Mahal et al., 2011). In particular, chronic prostatitis/chronic pelvic pain syndrome (CP/CPPS) accounts for nearly 90% of chronic prostatitis in males, and it affects nearly 10% of young men (Collins et al., 2002; Fall et al., 2010; Murphy et al., 2014). At present, the pathogenesis of CP/CPPS is still unclear, and the therapeutic efficiency of the treatment strategies that are recommended by clinical guidelines, such as α-blockers and non-steroidal anti-inflammatory drugs (NSAIDs), is still controversial (Fall et al., 2010). Therefore, new treatments are urgently needed.

Chinese herbalists who have contributed to traditional Chinese medicine (TCM) have a history spanning thousands of years of valued experience in fighting illnesses, and these approaches have achieved remarkable effects (Dai et al., 2020). In particular, many molecular components of TCM, such as quercetin and berberine, have significant anti-inflammatory effects in the treatment of inflammation (Zhang Y. et al., 2021; Shen et al., 2021). Ningmitai capsules have been used to treat CP/CPPS in the clinic for several years in China. Their therapeutic effect has been recognized at home and abroad. Its main ingredients are Touhualiao (Polygonum capitatum Buch.-Ham. ex D. Don), Baimaogen (Imperata cylindrica Beauv. Var. Major (Nees) C. E. Hubb.), Dafengteng (Cocculus orbiculatus (L.) DC.), Sankezhen (Berberis soulieana Schneid, Berberis wilsonae Hemsl, Berberis poiretii Schneid, and Berberis vernae Schneid.), Xianhecao (Agrimonia pilosa Ledeb.), Mufurongye (Hibiscus mutabilis L.), Lianqiao (Forsythia suspensa (Thunb.) Vahl), etc*.* (Cai and Shang, 2017; Jin et al., 2018; Zhang K. et al., 2021). Chen et al. found that Ningmitai capsules can significantly reduce the oxidative stress index of the prostate and attenuate substance P secretion by the dorsal root ganglia of rat models of experimental autoimmune prostatitis (EAP) (Chen et al., 2020).

However, the mechanism and targets by which Ningmitai functions in the treatment of CP/CPPS still need further exploration. In particular, the blood–prostate barrier can reduce the local drug concentration in the prostate, which reduces the therapeutic effect of the drug (Xia et al., 2021). *In vivo*, immune cells can penetrate the blood–testis barrier, and the blood–prostate barrier affects local immunity and improves the resolution of chronic inflammation (Pérez et al., 2014; Liu et al., 2021). In other words, targeting systemic immunity may influence local immunity through effects on immune cells. CP/CPPS is an autoimmune disease that has recently been widely studied by global researchers (Chen et al., 2021). Some studies have pointed out that the local infiltration of proinflammatory immune cells and the maintenance of inflammatory cells by inflammatory factors may be important causes of CP/CPPS (Alexander et al., 1998; Apkarian et al., 2006; Hsu et al., 2013; Lundh et al., 2013; Liu et al., 2020). TCM includes multiple anti-inflammatory and antioxidant components and has a wide range of targets related to chronic inflammation in the body. Can TCM improve local immunity by affecting systemic immunity?

Additionally, the inflammatory factor chemokine (C-C motif) ligand 2 (CCL2), which is related to pain, is increased in the prostates of CP/CPPS patients and animal models (Desireddi et al., 2008; Quick et al., 2012). CCL2, which is also called macrophage chemo-attractant protein-1 (MCP-1), is secreted by monocytes and can stimulate nerve endings to produce pain-afferent signals. CCL2 can recruit monocytes to accumulate in sites of inflammation to maintain and exacerbate inflammation (Quick et al., 2012; Jiang et al., 2020). In immune cells and tissue cells, the nuclear factor–kappa B (NF–κB) and mitogen-activated protein kinase (MAPK) pathways are activated by inflammatory stimulation, and these conditions can stimulate the secretion of CCL2, which in turn has a proinflammatory effect (Abbadie et al., 2009; Gao et al., 2009; Kobayashi et al., 2017; Meng et al., 2018).

In our study, based on the presence of a variety of anti-inflammatory and antioxidant Chinese medicines in Ningmitai capsules, according to the Encyclopedia of Traditional Chinese Medicine (ETCM) and Integrative Pharmacology-based Research Platform of Traditional Chinese Medicine (TCMIP) databases, we found that one of the major Ningmitai capsule ingredients, namely, quercetin, can inhibit CCL2 production in tissues, which may be related to MAPK-related proteins and pathways (Xu H. Y. et al., 2019). Then, we successfully established a NOD-EAP (non-obese diabetes-experimental autoimmune prostatitis) mouse model of CP/CPPS to investigate inflammation of the prostate. Next, after continuous gavage of the Ningmitai capsule solution into mice with CP/CPPS, Ningmitai capsules were absorbed through the gastrointestinal tract and distributed to the whole body by the bloodstream. We found that the proportions of systemic proinflammatory immune cells and local proinflammatory immune CD11b^+^Ly6C^hi^ cells were decreased *in vivo* through the NF–κB and MAPK pathways. This treatment further reduced the secretion of CCL2 by local immune cells in the prostate, improved inflammation, and reduced pelvic pain in mice with CP/CPPS.

## Materials and methods

### Traditional Chinese medicine

Ningmitai capsules were obtained from Guiyang Xintian Pharmaceutical Co., Ltd. According to the methods described in published studies, high-performance liquid chromatography (HPLC) chromatograms of the contents of effective components, such as quercetin, were analyzed using an Agilent 1260 High Performance Liquid Chromatograph, DAD Detector (Agilent Technologies Co., Ltd.) (Xu Y. et al., 2019; Zhang K. et al., 2021). This kind of antipyretic, analgesic, anti-inflammatory, and diuretic TCM has been widely used in clinical treatment, and Ningmitai capsules are included in the “Instructions for Clinical Medication of the People’s Republic of China Pharmacopoeia -Volume of Traditional Chinese Medicine Prescription (2015),” which was compiled by the National Pharmacopoeia Committee, and the “Guidelines for Diagnosis and Treatment of Urology and Andrology Diseases in China (2019),” which were set by the Chinese Urology Association of the Chinese Medical Association (Cai and Shang, 2017; Zhang K. et al., 2021). The gavage dosage that works best for treatment was determined based on a published article (1.80 g/kg/d, 0.10 g crude drug/ml) (Chen et al., 2020).

### Bioinformatics analysis of traditional Chinese medicine

According to the ETCM and TCMIP databases and the methods described in published articles (Xu H. Y. et al., 2019), the target genes and pathways related to the active ingredients of our TCM, as shown by the HPLC chromatograms, were explored and analyzed. A pathway/network/interaction analysis was performed according to the website http://www.tcmip.cn/ETCM/index.php/Home/Index/index.html. We can use the bioinformatics analysis function by following the website instructions and the website owner’s published article.

### Animals and model

Healthy 8-week-old male NOD/ShiLtJ mice were used for the experiments. The animals were purchased from Gempharmatech. The mice were housed in a sterile specific-pathogen-free (SPF) environment with 12 h of light and 12 h of darkness. The mice had free access to water and food. All the methods were approved by the Animal Care and Use Committee of Sun Yat-sen University (approval number: SYSU-IACUC-2022-000570). According to the American Veterinary Medical Association (AVMA) Guidelines for the Euthanasia of Animals: 2013 Edition, the animals were killed by anesthesia on the specified date.

The CP/CPPS animal model was established according to a previously published method (Rivero et al., 1998; Zhang L. G. et al., 2020). In this experiment, non-obese diabetic mice were used to establish the animal model because this model is an internationally recognized mouse model of chronic prostatitis; these mice were used to establish the chronic pelvic pain syndrome. That is, extracts of the male reproductive accessory gland (MAG) of male Wistar rats were mixed with an equal volume of complete Freund’s adjuvant (CFA) and subjected to repeated aspiration and mixing (the criterion for a successful mixing was that after the mixture of the MAG extract and CFA was dropped on the surface of pure water, it floated in a spherical shape). On day 0 and day 15, a mixture of the MAG extract and CFA was injected subcutaneously near the lymph nodes at four different sites on the shoulder, tail, and neck of the mouse body (25 μl per site).

### Pelvic pain behavioral testing

On days 30 and 60 after the first immunization, von Frey filaments were used to test the pain in the pelvis and the plantar area of the hind paw according to published methods (Laird et al., 2001; Quick et al., 2013). Five different filaments were used to test the frequency of positive responses, and 0.04, 0.16, 0.4, 1.0, and 4.0 g filaments were applied to the pelvis, lower abdomen, and paw. There are four behaviors of the von Frey test that are considered to be positive responses to filament stimulation: sharp abdominal contractions, immediate licking, scratching at the filament stimulation area, or jumping. The response frequency is calculated as a percentage of positive responses, and the data are reported as an average percentage of the response frequency. The 50% threshold was assessed by using the up and down method (Chaplan et al., 1994).

### Tissue preparations and histological analysis

Fresh tissue was harvested into Tissue-Tek optimum cutting temperature (OCT) complex and frozen in a −80 freezer. Next, 5-μm thick tissue sections were prepared and placed on glass slides (Bio-Optical). The sections were blocked with Tween supplemented with 0.5% FBS and 0.05% sodium thiobarbital for 30 min and incubated with a primary antibody or IgG control antibody overnight at 4°C. Then, the cells were incubated with a secondary antibody at 37°C for 60 min. Finally, the cells were stained with 4′,6-diamidino-2-phenylindole (DAPI) for 5 min at room temperature. Immunofluorescence (IF) signals were imaged using a laser scanning confocal microscope (LSM780, Zeiss, Germany). For hematoxylin–eosin (HE) staining, tissues were fixed in 4% PFA (Sigma) overnight, serially dehydrated and embedded in paraffin. Then, sections with a thickness of 5 μm were placed on glass slides. The samples were stained with an HE staining kit (Servicebio). The antibodies were as follows: fluorescent primary antibody: MCP1/CCL2 (5H2) Mouse mAb (ZENBIO (220691), 1:100) and CD11b Rabbit pAb (ZENBIO (380675), 1:200). The fluorescent secondary antibodies were anti-rabbit IgG (Fluor^®^ 555 Conjugate, CST (4,413), 1:400) and anti-mouse IgG (Fluor^®^ 488 Conjugate, CST (4,408), 1:400).

### Mouse inflammatory cytokine measurement

The LEGENDplex™ Mouse Inflammation Panel was used to measure the levels of the IL-1α, IL-1β, IL-6, IL-10, IL-12p70, IL-17A, IL-23, IL-27, MCP-1, IFN-β, IFN-γ, TNF-α, and GM-CSF cytokines in the mouse samples. Data acquisition was performed according to the instructions at biolegend.com/en-us/legendplex, and FCS files were analyzed using BioLegend’s LEGENDplex™ data analysis software.

### Western blotting

Extracted fresh animal tissues or cells were triturated in liquid nitrogen or sonicated with RIPA lysis buffer (Millipore)-supplemented protease inhibitors. After centrifugation at 15,000 × g for 10 min at 4°C, the protein concentrations were measured by BCA, and the protein concentrations were adjusted for loading onto gels (Sigma). The solutions were loaded on SDS–polyacrylamide gels for electrophoresis, and the proteins were transferred to 0.22-µm polyvinylidene fluoride (PVDF) membranes (Millipore). The membranes were blocked with 7% bovine serum albumin for 60 min at room temperature and incubated with the indicated primary antibodies overnight at 4°C, followed by incubation with secondary antibodies for 1 h at room temperature. A chemiluminescence kit (Millipore) was used to detect the target bands. Western blot antibody information is presented in Table 1.

**TABLE 1:** Antibody information used in the article.

Primary antibody		
MCP1/CCL2 (5H2) Mouse mAb	1:1,000(WB)	ZENBIO(220691)
GAPDH Rabbit Ab	1:3,000(WB)	Affinity (AF7021)
P-STAT3 Rabbit Ab	1:1,000(WB)	CST (9,131)
STAT3 Mouse Ab	1:1,000(WB)	CST (9,139)
p-P65 Rabbit Ab	1:1,000(WB)	CST (3,031)
P65 Rabbit Ab	1:1,000(WB)	CST (8,242)
p-JNK Rabbit Ab	1:1,000(WB)	CST (4,668)
JNK Rabbit Ab	1:1,000(WB)	CST (9,252)
p-P38 Rabbit Ab	1:1,000(WB)	CST (4,511)
P38 Rabbit Ab	1:1,000(WB)	CST (8,690)
p-ERK Rabbit Ab	1:1,000(WB)	CST (4,370)
ERK Rabbit Ab	1:1,000(WB)	CST (4,695)
Secondary antibody		
Anti-mouse IgG HRP-linked Ab	1:5,000	CST (7,076)
Anti-rabbit IgG HRP-linked Ab	1:5,000	CST (7,074)

### Measurement of superoxide dismutase, malondialdehyde, and GSH levels

After the mice were sacrificed, the mouse prostate tissues were homogenized and then placed in phosphate-buffered saline (PBS). The homogenates were centrifuged at 2,500 rpm/min for 10 min at 4°C, and the supernatants were extracted for further analysis of oxidative stress levels. Superoxide dismutase (SOD) and malondialdehyde (MDA) contents were measured according to the corresponding detection kits (Nanjing Jiancheng Bioengineering Institute). The data were further analyzed according to the instructions to calculate the protein content.

### Flow cytometry

Fresh tissue was crushed into small pieces and shaken slowly with Liberase TL (100 U/ml; Roche) and DNase I (100 U/ml; Sigma) in a 37°C incubator for approximately 20 min. Blood samples were lysed with erythrocyte lysis buffer (BioLegend) on ice at 4°C for 15 min. Subsequently, the cell suspensions were passed through a 70-μm cell filter to generate single-cell suspensions. Single-cell suspensions, tissue samples, and blood samples were incubated with appropriate antibodies (Table 2) for 15 min in the dark. The cells were washed twice, resuspended in flow assay tubes with PBS, and analyzed by using MoFlo Astrios EQ (Beckman Coulter) for at least three independent experiments. The data were analyzed by using FlowJo V10.0 (Tree Star). The antibody information is listed in Table 2.

**TABLE 2:** Antibody information used in the article.

Antibodies used in flow cytometry		
CD11b-PE/CY7 Antibody	1:100	BioLegend (101215)
Ly6C-APC Antibody	1:100	BioLegend (128015)

### Statistical analysis

Comparisons between groups were performed using a *t*-test or one-way analysis of variance (ANOVA), and Tukey’s test was used to compare multiple groups. All the experiments were performed with a minimum of three replicates, and differences were considered statistically significant when *p* < 0.05 (*p < 0.05; **p < 0.01). Statistical analysis was performed using GraphPad Prism 8.0 software, and statistical graphs were generated. Error bars represent the standard error of the mean.

## Results

### Bioinformatics analysis of quercetin, the main ingredient of Ningmitai capsules, with the ETCM and TCMIP databases

**FIGURE 1:**
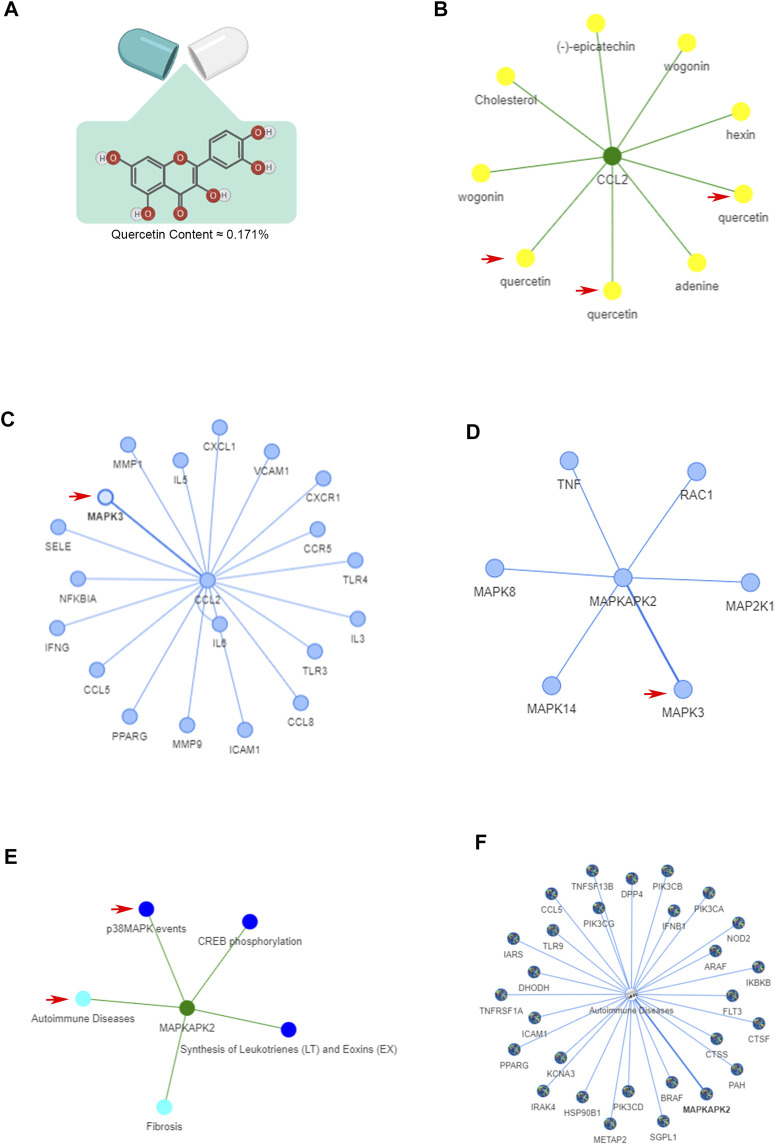
Bioinformatics analysis of quercetin (the main ingredient of Ningmitai capsules) in ETCM and TCMIP databases. **(A)** Structural formula and content of quercetin in Ningmitai capsules. **(B)** Target–ingredient interaction of CCL2 and quercetin in the ETCM database. **(C)** Protein‒protein interaction of the CCL2 gene with other genes. **(D)** MAPK protein‒protein interactions. **(E)** Target–pathway–disease interaction of the MAPK pathway with autoimmune diseases. **(F)** Pathway–disease interaction of the MAPK pathway with autoimmune diseases.

The structural formula and content of quercetin (≈0.171%) were based on previous results of HPLC analysis with an Agilent 1260 High Performance Liquid Chromatograph, DAD Detector (Agilent Technologies Co., Ltd.) (Xu Y. et al., 2019) are shown in Figure 5A. The bioinformatics analysis of the ETCM database was performed to assess key molecules and pathways related to the pain-related factor CCL2 and quercetin. As shown in Figure 1B, CCL2 was most strongly influenced by quercetin in the ETCM database. From the network, we found that another ingredient, wogonin, in Ningmitai capsules can also affect CCL2 secretion. The involvement of the MAPK signaling pathway, which is the upstream pathway of CCL2, was confirmed by the ETCM database and previous articles (Figures 1C–E). In addition, autoimmune responses are commonly accepted as causes of CP/CPPS in the current literature, and the MAPK signaling pathway was significantly connected with this kind of disease (Figure 1F).

### Establishment of the chronic prostatitis/chronic pelvic pain syndrome mouse model

**FIGURE 2:**
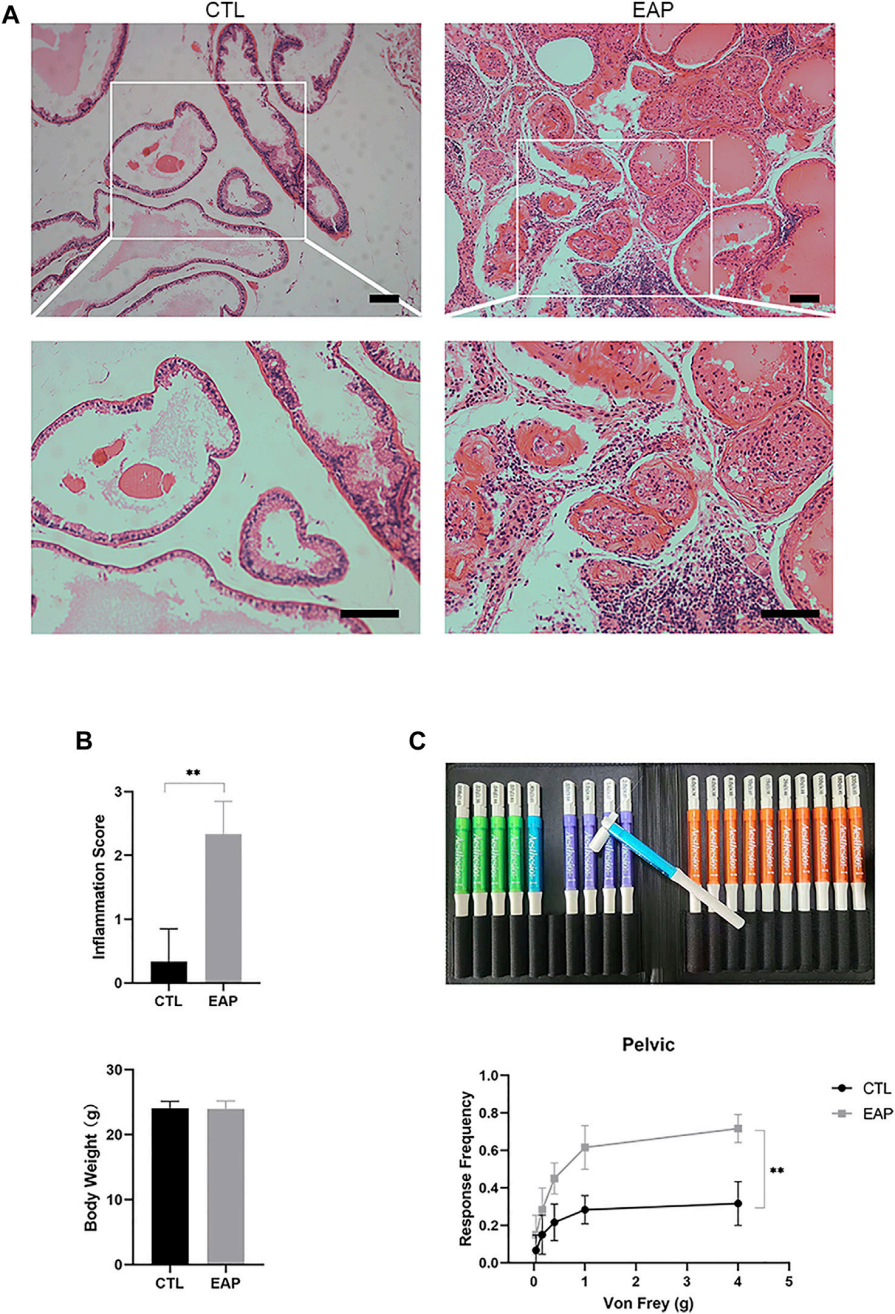
Successful establishment of the CP/CPPS mouse model. **(A)** Sections of prostate tissues were stained with HE. Scale bar = 50 μm; CTL = control; EAP = experimental autoimmune prostatitis mice. **(B)** Inflammatory score and weight in the CTL and EAP groups. n = 6. **(C)** The Von Frey filament response frequency test in the CTL and EAP groups. n = 6. (*: *p* < 0.05; **: *p* < 0.01)

The most widely accepted animal model of CP/CPPS (NOD-EAP) was established as described in the Methods section; in this model, the tissue structure of the mouse prostate changed after the model was successfully established, and a large number of inflammatory cells infiltrated the prostate (Figure 2A). According to a previously published article (Liu et al., 2021), the inflammatory score was used to assess the inflammatory state, and it was found that the prostate tissue inflammatory score was significantly increased in the EAP group (*p* < 0.001), but the difference in body weight between the two groups was not statistically significant (*p* > 0.05) (Figure 2B). Von Frey pain behavior test filaments were used to measure the pain of the mice with EAP, and we found that the response frequency was significantly increased in mice with EAP (*p* = 0.002) (Figure 2C). In this part of the experiment, the mouse CP/CPPS model was shown to have been successfully established, and it was ready for our subsequent research.

After Ningmitai treatment, prostatic inflammation and inflammatory pain-related pathway activity in mice decreased.

**FIGURE 3:**
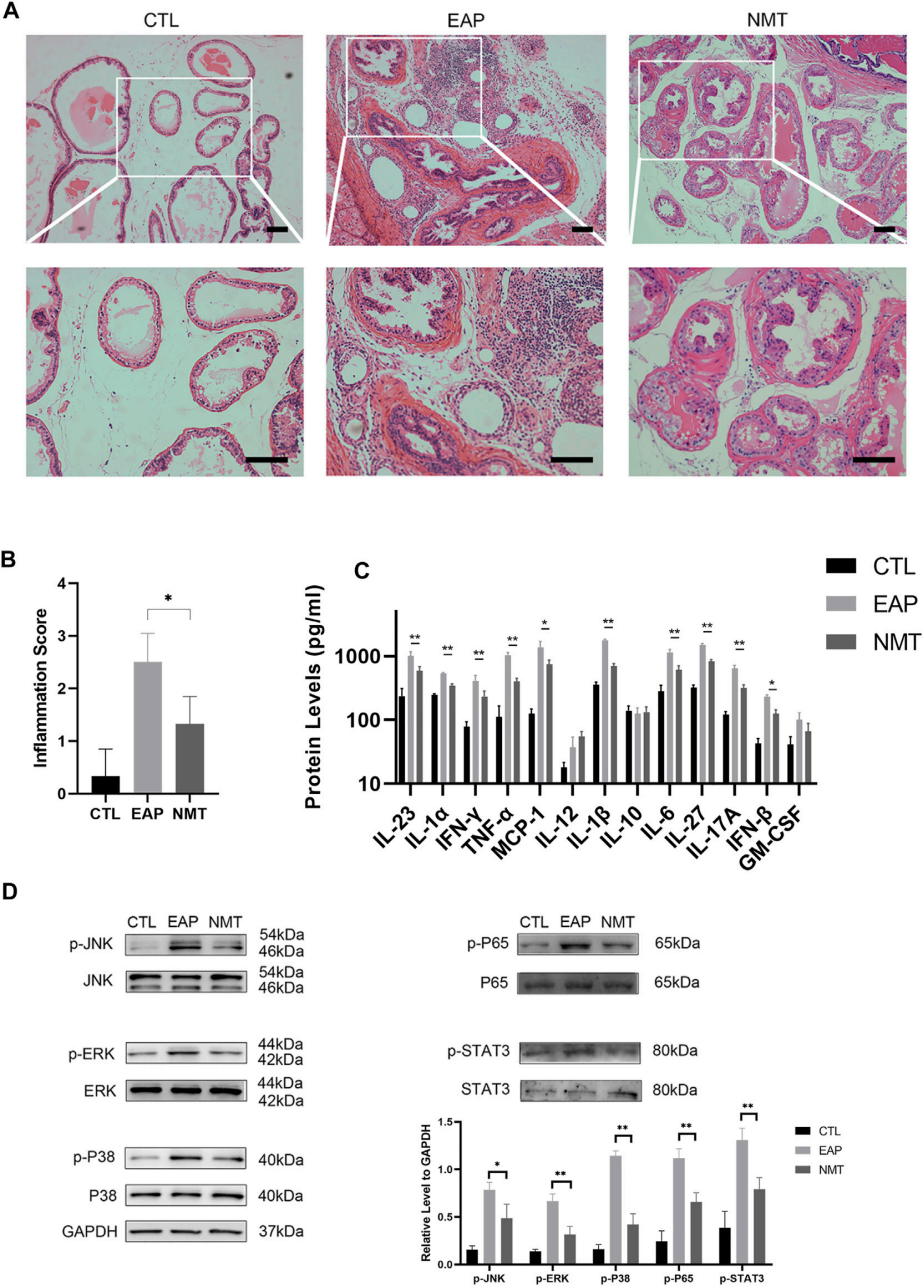
Decreased prostatic inflammation and inflammatory pain-related pathway activation in mice. **(A)** Sections of prostate tissues were stained with HE. Scale bar = 50 μm; CTL = control; EAP = experimental autoimmune prostatitis mice; NMT: Ningmitai-treated mice. **(B)** Inflammatory scores of the CTL, EAP, and NMT groups. n = 6. **(C)** Thirteen inflammatory-related factors in the CTL, EAP, and NMT groups. n = 6. **(D)** Western blotting analyzing of p-JNK, JNK, p-ERK, ERK, p-P38, P38, p-P65, P65, p-STAT3, STAT3, and GAPDH expressions as well as a statistical analysis of the results in the CTL, EAP, and NMT groups. n = 6. (*: *p* < 0.05; **: *p* < 0.01)

Inflammatory pain factor-related pathways and inflammation-related pathways were the focus of this experiment. Figure 3A shows the tissue structure of the prostate in the three groups of mice. The inflammation of the NMT group was significantly decreased. After the Ningmitai capsule treatment, the prostate inflammatory score of the mice in the treatment group was significantly lower than that of the mice in the model group (*p* = 0.03) (Figure 3B). An assessment of thirteen inflammatory-related factors showed that local inflammation in the prostate of mice was significantly reduced after treatment (Figure 3C). The expression of components of inflammatory pain-related pathways and the inflammation-related pathways MAPK and NF–κB decreased, as shown by Western blotting analysis of the expressions of p-JNK (*p* = 0.014), JNK, p-ERK (*p* < 0.001), ERK, p-P38 (*p* < 0.001), P38, p-P65 (*p* < 0.001), P65, p-STAT3 (*p* < 0.001), and STAT3 (Figure 3D).

### Pelvic pain relief in mice with chronic prostatitis/chronic pelvic pain syndrome after Ningmitai treatment

**FIGURE 4:**
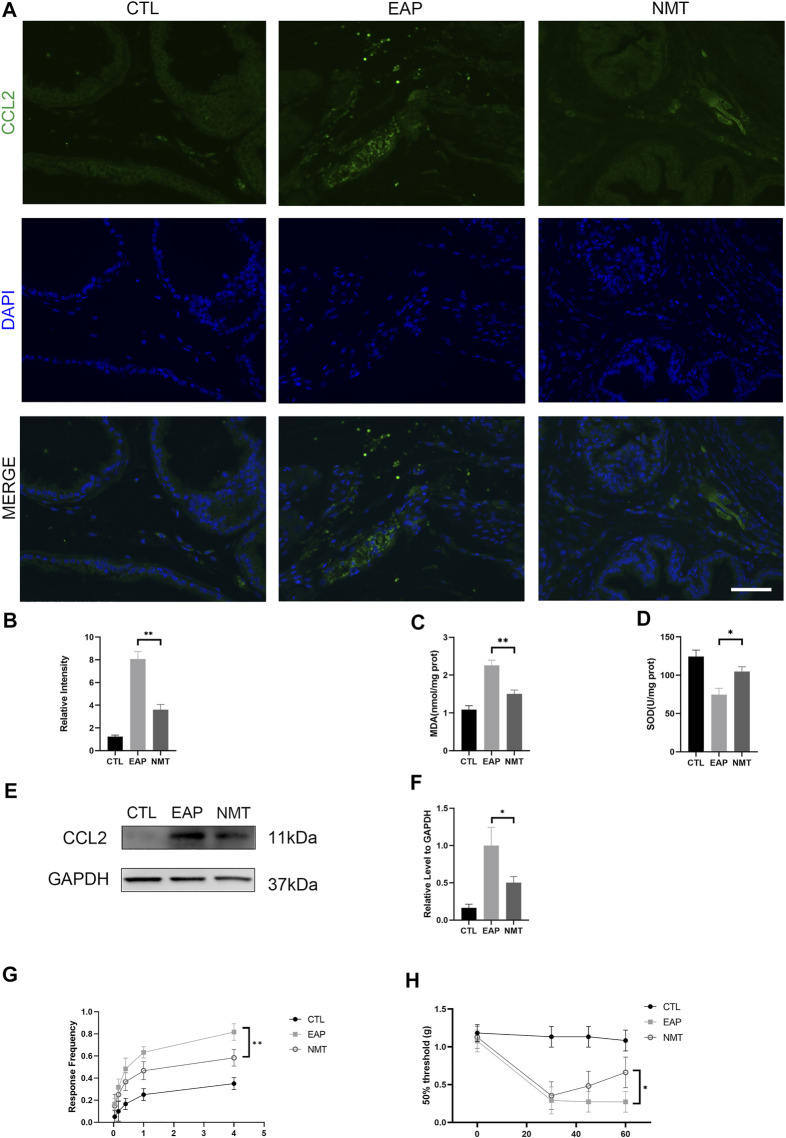
Pelvic pain relief and oxidative stress reduction in mice with CP/CPPS after Ningmitai treatment. **(A)** Immunofluorescence experiments showing the expression of the pain-related factor CCL2. Scale bar = 50 μm. **(B)** Immunofluorescence experiments showing the relative intensity in the CTL, EAP, and NMT groups. n = 6. CTL = control; EAP = experimental autoimmune prostatitis mice; NMT: Ningmitai-treated mice. **(C–D)** The activities of SOD and MDA in the CTL, EAP, and NMT groups were measured by kits. n = 6. **(E–F)** Western blotting analysis of CCL2 and GAPDH expressions as well as statistical graphs of the results in the CTL, EAP, and NMT groups n = 6. **(G)** Von Frey filament response frequency test in the CTL, EAP, and NMT groups n = 6. **(H)** The Von Frey filament up and down test revealed a 50% threshold between the CTL, EAP, and NMT groups. n = 6. (*: *p* < 0.05; **: *p* < 0.01)

In the study of pain, we used IF experiments to measure the expression of the prostatitis pain-related factor CCL2, and its expression was significantly decreased after treatment (*p* < 0.001) (Figures 4A,B). Moreover, as important downstream proteins of MAPK, the SOD and MDA contents indicate the level of oxidative stress. After treatment, we found that the level of local oxidative stress in the prostate significantly decreased (*p* = 0.002) (*p* = 0.015) (Figures 4C,D). The expression of the inflammatory pain chemokine CCL2 was decreased after treatment, as shown by Western blotting (*p* < 0.001) (Figures 4E,F). Von Frey pain detection filament showed that the response frequency was significantly decreased (*p* = 0.041) and that the 50% threshold of mice after treatment was significantly improved (*p* = 0.025) (Figures 4G,H).

### Changes in the systemic and local immune responses in mice after Ningmitai treatment

**FIGURE 5:**
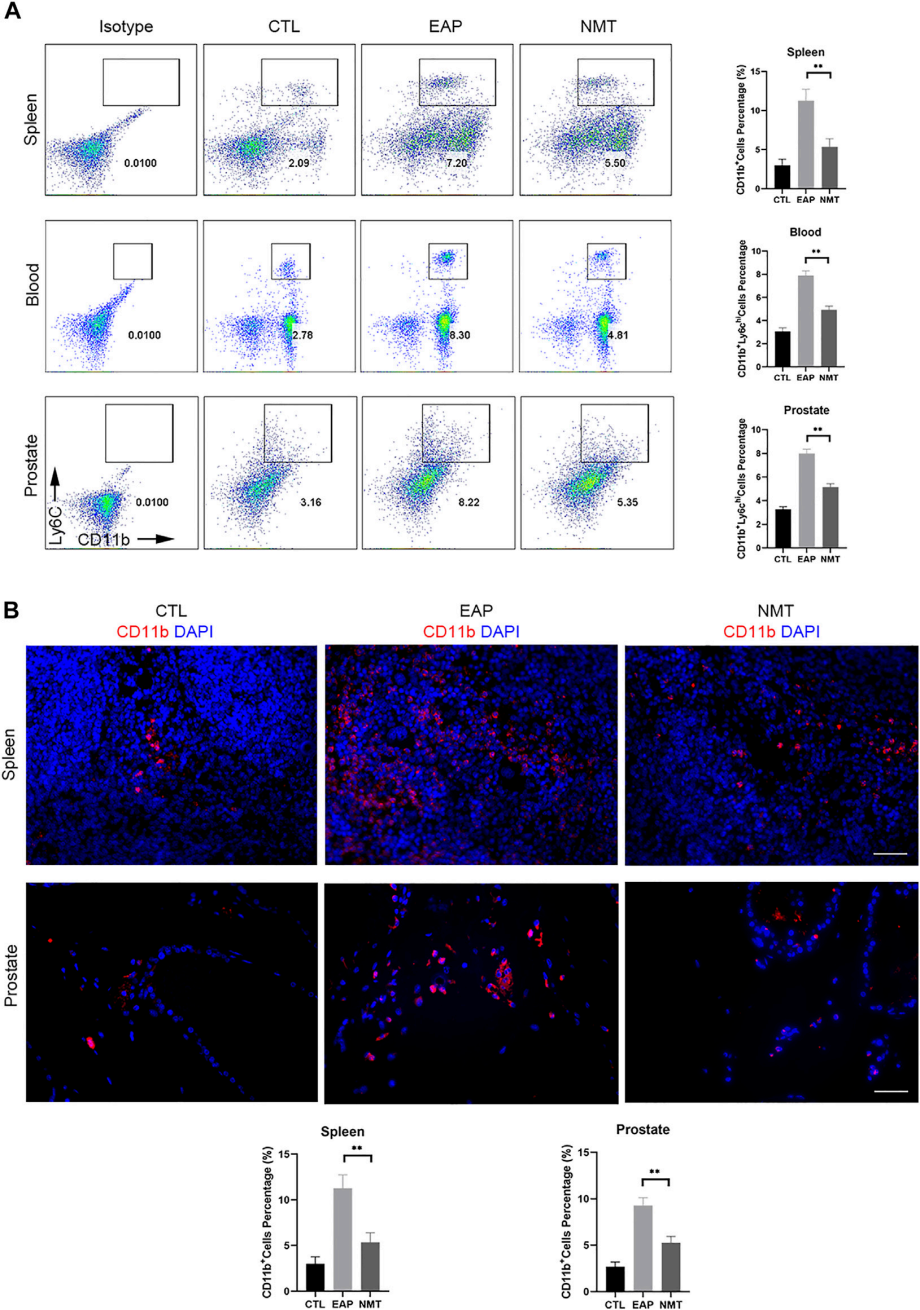
Changes in systemic and local immune responses in mice after Ningmitai treatment. **(A)** Flow cytometry analysis showing the proportion of CD11b^+^Ly6C^high^ immune cells in the spleen, blood, and prostate, and statistical analysis of the results in the CTL, EAP, and NMT groups n = 6. CTL = control; EAP = experimental autoimmune prostatitis mice; NMT: Ningmitai-treated mice. **(B)** Immunofluorescence experiments showed the expression of CD11b^+^ immune cell infiltration and statistical analysis of the results in the CTL, EAP, and NMT groups. n = 6. (*: *p* < 0.05; **: *p* < 0.01)

**FIGURE 6:**
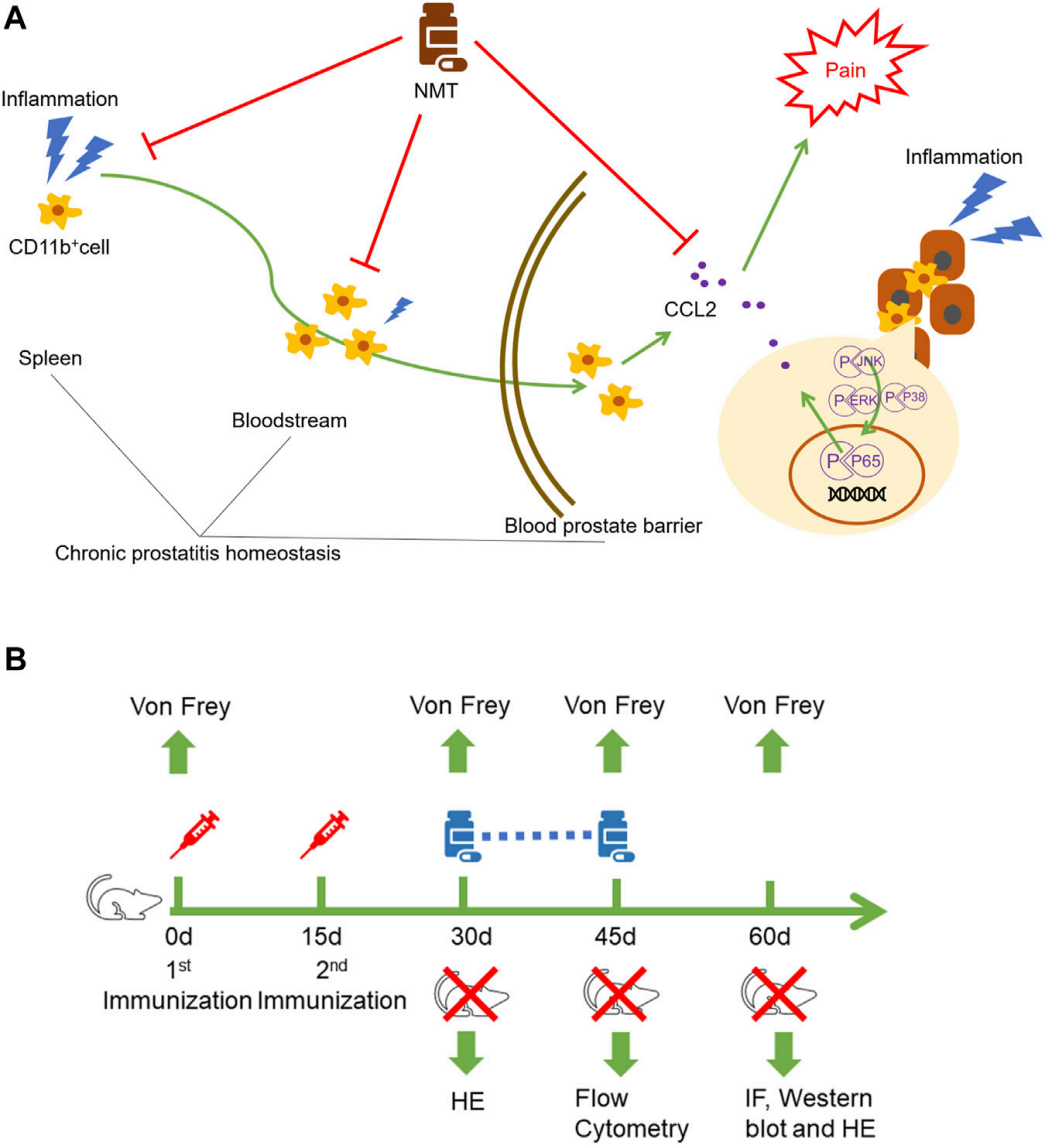
Schematic diagram and experimental flow chart. **(A)** Ningmitai capsules affect local immune cells by modulating the CD11b^+^ immune cells in the systemic and local immune responses and reduce the immune cell secretion of the inflammatory factor CCL2 by inhibiting the MAPK, NF–kB, and STAT3 pathways. **(B)** EAP models were established by two injections on days 0 and 15. On the 30th day, behavioral tests (Von Frey) and HE staining were performed to determine whether the model was successfully established. Gavage treatments were started on the day when the model was successfully established, and gavage treatment was carried out for 15 consecutive days. Behavioral tests and flow cytometry analysis were performed on the 45th day. Finally, pain behavioral tests, immunofluorescence, Western blotting, and HE staining were performed on the 60th day.

We mentioned that immune cells can migrate to tissues and play proinflammatory and anti-inflammatory roles at the site of inflammation. We found that the proportions of CD11b^+^Ly6C^high^ immune cells in the spleen, blood, and prostate decreased in mice after drug administration (Figure 5A). IF experiments showed that CD11b^+^ immune cell infiltration was decreased in the spleen and prostate of the mice after treatment (Figure 5B). This showed that in the autoimmune disease model, TCM has a therapeutic effect on both systemic immunity and local immunity in mice. TCM affects the local environment by affecting the overall immune environment of the mouse, and it allows immune cells to play an anti-inflammatory role in fighting against inflammation. The schematics of the underlying mechanism described in this article (Figure 6A) and the experimental flow chart of the article (Figure 6B) are shown.

## Discussion

At present, clinical guidelines are more inclined to recommend well-established treatment for CP/CPPS (Pontari, 2003). Anti-inflammatory drugs, α-blockers, or α-adrenergic receptor antagonists and antibiotics are widely used in clinical practice, but their effects are still controversial (Magistro et al., 2016). Therefore, more efficient treatments, awareness, and understanding of CP/CPPS are urgently needed.

TCM has been widely used in the clinical trials of CP/CPPS in recent years. The treatment of CP/CPPS through the application of the unique diagnosis and treatment system of TCM has achieved significant therapeutic effects (Liao et al., 2011; Jin et al., 2018; Zhang W. J. et al., 2020). Studies have shown that the ingredients of TCM can improve the peripheral blood supply and regulate bodily function through multi-faceted and multi-targeted mechanisms related to anti-inflammatory (STAT3, NF–κB) and anti-oxidative stress (MAPK) pathways (Song et al., 2013; Wu et al., 2015; Gong et al., 2020). At present, an increasing number of researchers believe that CP/CPPS is a kind of autoimmune disease, which is one of the reasons why the NOD-EAP mouse model used in this research is widely accepted by researchers around the world. Researchers currently believe that autoimmune disorders that involve local and systemic immunities and lead to chronic inflammation of the prostate and pelvis cause CP/CPPS. There are no reports about proteins or pathways that are related to CP/CPPS and targeted by TCM according to the Chinese medicine database; however, through network bioinformatics analysis of the TCM database, we found a network association between the inflammatory CCL2–MAPK pathway and autoimmune diseases. The multi-target and multi-mechanism principle of the anti-inflammatory effects of TCM prompts researchers to conduct a thorough research on some of the ingredients of TCM. Our TCM network analysis data showed that quercetin, which is one of the components of Ningmitai capsules, can inhibit the inflammatory protein CCL2, which is related to pain, by targeting MAPK-related proteins and pathways, according to the bioinformatics analysis. Moreover, some researchers also revealed the advantages of quercetin in the treatment of CP/CPPS (Meng et al., 2018). Therefore, we think that Ningmitai capsules may alleviate CP/CPPS *via* the effects of quercetin on the CCL2–MAPK pathway, and we will perform the necessary research to certify this theory.

In addition, numerous clinical and empirical medicine studies have shown that TCM has a strong effect in the treatment of CP/CPPS, and patients can achieve satisfactory results to a certain degree (Zhang et al., 2007). However, due to the limitations related to the clinical sample size and the single center included in the study, the unclear etiology and mechanism of CP/CPPS, and the numerous targets of Chinese herbal medicine, evidence-based approaches have not sufficiently proven the efficacy of this treatment (Chen et al., 2020). Therefore, the efficacy and safety of TCM in the treatment of CP/CPPS still require further in-depth exploration, including exploration of its therapeutic mechanism and targets, to provide sufficient evidence for its clinical application. In this study, we focused on one ingredient from Ningmitai capsules and found a potential target and pathway by analyzing the database. Our subsequent study successfully confirmed that MAPK pathway activation, NF–κB pathway activation, and target protein CCL2 expression were decreased after treatment.

With the aim of ameliorating autoimmune disorders related to local and systemic immunities, we studied the changes in the immune cells in the local prostate and systemic environments of mice with EAP, especially the changes in the CD11b^+^ mononuclear cell/macrophage populations, which are of great significance for the study of prostate inflammation and pelvic pain (Roca et al., 2009). Systemic and local immunity exist in homeostasis; moreover, CD11b^+^ monocytes surveil and migrate freely in the spleen, blood, and local tissues, such as the prostate. Most researchers believe that the role of the blood–prostate barrier leads to a decrease in the effects of drug treatment. Therefore, improving local immunity *via* treatment with drugs that affect systemic immunity may be another treatment strategy for TCM. When inflammation occurs, such monocytes acquire a CD11b^+^Ly6C^high^ phenotype, and these cells are proinflammatory cells that migrate to the site of inflammation, playing a role in exacerbating and maintaining inflammation. Conversely, to control and eliminate inflammation, CD11b^+^Ly6C^low^ monocytes target and migrate to sites of inflammation and play roles in the resolution of inflammation (Swirski et al., 2007; Swirski et al., 2009). Based on our CP/CPPS model and previously published studies, we found that inflammation in the mouse prostate was more severe, and we observed higher proportions of proinflammatory CD11b^+^Ly6C^high^ monocytes/macrophages in the lung, spleen, and blood of the mice. This may indicate that the systemic immunity of the mice was changed by inflammation (Liu et al., 2021). As we mentioned previously, CD11b^+^ immune cells patrol the body and play a proinflammatory or anti-inflammatory role. If we can alter the phenotype of the immune cells in the system to an anti-inflammatory phenotype, then these cells could migrate to the local site of inflammation and resolve this local inflammation. Based on this, we learned that although the presence of the blood–prostate barrier may affect the efficacy of drug therapy in mice, we ameliorated the activity of the immune cells in the mice and improved the systemic immune responses by gavage with TCM. These effects may contribute to the transformation of immune cells into an anti-inflammatory phenotype, and inhibiting proinflammatory phenotypes may lead to better treatment of local inflammation in the prostate. We found that the proportion of CD11b^+^Ly6C^high^ cells, which are proinflammatory monocytes in the immune system of mice, was decreased after treatment by gavage. With decrease in the proportion of CD11b^+^Ly6C^high^ immune cells in the spleen and blood, the migration of these proinflammatory cells to the prostate decreased, the local proportion of proinflammatory immune cells decreased, and inflammation in the prostate was ameliorated.

According to previous studies, we know that the inflammatory chemokine CCL2, which is related to pain, is mainly secreted by immune cells and greatly contributes to the recruitment of blood monocytes to sites of inflammation (Deshmane et al., 2009; Yoshimura, 2018). When inflammation occurs, local inflammation is more severe. The activation of the NF–κB pathway, MAPK pathway, and some other pathways lead to the increased secretion of the downstream protein CCL2 by immune cells such as monocytes or macrophages (Deshmane et al., 2009). Increased CCL2 levels continue to recruit monocytes/macrophages, maintaining and exacerbating inflammation. In addition, CCL2, which is an inflammatory chemokine related to pain, also stimulates peripheral nerve receptors, leading to the generation of pain sensation (Wang et al., 2020). After gavage treatment, we measured the levels of the components of the upstream CCL2 pathway and the CCL2 protein and found that the expression levels were decreased in the prostates of mice after treatment. The Von Frey pain test also revealed that pelvic pain was reduced in these mice. Based on our previous studies, the secretion of substance P by the dorsal root ganglia can be inhibited by Ningmitai capsules in rats with CP/CPPS (Chen et al., 2020). These results confirmed that Ningmitai capsules can significantly ameliorate pelvic pain in mice with CP/CPPS.

## Conclusion

In this study, we found that one of major ingredients of Ningmitai capsules, quercetin, can reduce the secretion of CCL2 by inhibiting the MAPK, NF–kB, and STAT3 pathways, thus alleviating pain, enhancing the resolution of inflammation, and reducing oxidative stress in mice with CP/CPPS. Moreover, we found that the proportion of CD11b^+^Ly6C^high^ proinflammatory immune cells in the systemic and local immune responses of mice was decreased after Ningmitai capsule treatment. The infiltration of CD11b^+^ immune cells into the spleen (systemic immune organ) and prostate (local immune site) was reduced, which proved that Ningmitai capsules can treat mice with CP/CPPS by affecting systemic and local immunities.

## Data Availability

The raw data supporting the conclusion of this article will be made available by the authors, without undue reservation.
