# Supplementary material for: Ningmitai capsules have anti-inflammatory and pain-relieving effects in the chronic prostatitis/chronic pelvic pain syndrome mouse model through systemic immunity
Source: Front Pharmacol. 2022 Oct 3;13:949316. doi: 10.3389/fphar.2022.949316 (PMC9574058; doi:10.3389/fphar.2022.949316)
Supplement: Supplementary file 1 [file Table1.DOCX]

| **Primary antibody:** |  |  |
| --- | --- | --- |
| MCP1/CCL2 (5H2) Mouse mAb | 1:1000(WB) | ZENBIO(220691) |
| GAPDH Rabbit Ab | 1:3000(WB) | Affinity (AF7021) |
| P-STAT3 Rabbit Ab | 1:1000(WB) | CST (9131) |
| STAT3 Mouse Ab | 1:1000(WB) | CST (9139) |
| p-P65 Rabbit Ab | 1:1000(WB) | CST (3031) |
| P65 Rabbit Ab | 1:1000(WB) | CST (8242) |
| p-JNK Rabbit Ab | 1:1000(WB) | CST (4668) |
| JNK Rabbit Ab | 1:1000(WB) | CST (9252) |
| p-P38 Rabbit Ab | 1:1000(WB) | CST (4511) |
| P38 Rabbit Ab | 1:1000(WB) | CST (8690) |
| p-ERK Rabbit Ab | 1:1000(WB) | CST (4370) |
| ERK Rabbit Ab | 1:1000(WB) | CST (4695) |
| **Secondary antibody:** |  |  |
| Anti-mouse IgG HRP-linked Ab | 1:5000 | CST (7076) |
| Anti-rabbit IgG HRP-linked Ab | 1:5000 | CST (7074) |

Table 1
