# Supplementary material for: Ningmitai capsules have anti-inflammatory and pain-relieving effects in the chronic prostatitis/chronic pelvic pain syndrome mouse model through systemic immunity
Source: Front Pharmacol. 2022 Oct 3;13:949316. doi: 10.3389/fphar.2022.949316 (PMC9574058; doi:10.3389/fphar.2022.949316)
Supplement: Supplementary file 2 [file Table2.DOCX]

Table 2

| **Antibodies used in flow cytometry** |  |  |
| --- | --- | --- |
| CD11b-PE/CY7 Antibody | 1:100 | BioLegend (101215) |
| Ly6C-APC Antibody | 1:100 | BioLegend (128015) |
